# Simulation to optimize the laboratory diagnosis of bacteremia

**DOI:** 10.1128/spectrum.01449-24

**Published:** 2024-09-24

**Authors:** Alessandro Gerada, Gareth Roberts, Alex Howard, Nada Reza, Anoop Velluva, Conor Rosato, Peter L. Green, William Hope

**Affiliations:** 1 Antimicrobial Pharmacodynamics and Therapeutics Group, Pharmacology Department, Institute of Systems, Molecular & Integrative Biology, University of Liverpool, Liverpool, United Kingdom; 2 Department of Infection and Immunity, Liverpool Clinical Laboratories, Clinical Support Services Building (CSSB), Liverpool University Hospitals NHS Foundation Trust, Liverpool, United Kingdom; 3 School of Engineering, Foundation Building, University of Liverpool, Liverpool, United Kingdom; MultiCare Health System, Tacoma, Washington, USA

**Keywords:** clinical microbiology, bloodstream infections, blood culture, discrete event simulation

## Abstract

Blood cultures are central to the management of patients with sepsis and bloodstream infection. Clinical decisions depend on the timely availability of laboratory information, which, in turn, depends on the optimal laboratory processing of specimens. Discrete event simulation (DES) offers insights into where optimization efforts can be targeted. Here, we generate a detailed process map of blood culture processing within a laboratory and use it to build a simulator. Direct observation of laboratory staff processing blood cultures was used to generate a flowchart of the blood culture laboratory pathway. Retrospective routinely collected data were combined with direct observations to generate probability distributions over the time taken for each event. These data were used to inform the DES model. A sensitivity analysis explored the impact of staff availability on turnaround times. A flowchart of the blood culture pathway was constructed, spanning labeling, incubation, organism identification, and antimicrobial susceptibility testing. Thirteen processes in earlier stages of the pathway, not otherwise captured by routinely collected data, were timed using direct observations. Observations revealed that specimen processing is predominantly batched. Another eight processes were timed using retrospective data. A simulator was built using DES. Sensitivity analysis revealed that specimen progression through the simulation was especially sensitive to laboratory technician availability. Gram stain reporting time was also sensitive to laboratory scientist availability. Our laboratory simulation model has wide-ranging applications for the optimization of laboratory processes and effective implementation of the changes required for faster and more accurate results.

**IMPORTANCE:**

Optimization of laboratory pathways and resource availability has a direct impact on the clinical management of patients with bloodstream infection. This research offers an insight into the laboratory processing of blood cultures at a system level and allows clinical microbiology laboratories to explore the impact of changes to processes and resources.

## INTRODUCTION

The prompt collection of blood cultures followed by empirical antimicrobial therapy is a standard of care for the management of patients with suspected sepsis (1–3). The results from blood cultures are used to make a raft of clinical decisions 24–48 h post clinical presentation. Hence, the laboratory diagnosis of bacteremia is paramount for patient care and is central to the effective functioning of tertiary healthcare systems. There has been extensive technical innovation in terms of assay development, automated incubation, and detection of microbial growth in blood cultures—these efforts have resulted in progressive improvements in the analytical sensitivity, time to positivity, and overall yield. In contrast, there has been relatively little attention to the broader system attributes underpinning the laboratory diagnosis of bacteremia, which is a complex, resource intensive, intricate, multi-step, and multi-user process.

Healthcare systems—like blood culture processing—are complex. They are composed of many components that sequentially interact to ultimately deliver patient care (4). Changes in process or resource availability often have a non-linear and seemingly unpredictable relationships with the overall performance of the healthcare system—thus, the probability of causing unintentional harm is relatively high (5). Simulation modeling, such as agent-based modeling (ABM) and discrete event simulation (DES), allows a deeper understanding of complex systems. While ABM is particularly suited to epidemiological transmission modeling, as evidenced by its extensive use in the SARS-CoV-2 pandemic (6, 7), DES is usually applied to systems composed of pathways, processes, and queueing where resources are limited (8). The latter is typical of many processes in healthcare for which the processing of blood cultures is an exemplar.

Well-calibrated discrete event simulators can be used to shorten test turnaround times (9), identify and reduce lost time (10), and plan for the impact of new laboratory health information systems (11). All of these events are extraneous to the intrinsic performance of a test. Here, we construct a simulation of blood culture specimens as they are collected from patients, transferred to the laboratory, analyzed, and reported. Our approach captures real-world practices that may deviate from standard operating procedures and miss other subprocesses that are relevant to overall performance (12). The simulator enables the impact of extraneous factors (e.g., staffing, work schedules) on blood culture performance to be modeled.

## MATERIALS AND METHODS

### Study setting

This study was conducted in a microbiology laboratory in Liverpool, UK, serving two acute hospital sites and multiple smaller specialist hospitals (total ~2,000 inpatient beds). The laboratory processes approximately 35,000 blood cultures annually, using automated incubators (BACTEC, BD, Maryland, USA) for incubation, Sepsityper and MALDI-TOF (Bruker Daltonics, Massachusetts, USA) for organism identification, and disk susceptibility for routine antimicrobial susceptibility testing.

### Process mapping

A qualitative blood culture laboratory process map was developed. Direct observations of the laboratory pathway were conducted by a single laboratory scientist over 3 days. Observations were recorded in a standardized form (see Fig. S1). The observer did not interact with the staff member performing the task. A flowchart was constructed in Microsoft Visio, similar to methods described by Lodemann et al. (13) and following ISO 5807:1985 (14).

Broadly, two grades of staff were involved in the blood culture laboratory pathways: laboratory technicians who mainly perform processing steps (e.g., labeling, loading onto incubators) and laboratory scientists, who mainly perform analytical steps (e.g., Gram stain interpretation).

### Direct observation of event timings

Further direct observation over a three-week period was used to populate the qualitative process maps with the time taken to complete events. Event start and end times were collected using automatically generated timestamps in Microsoft Excel. Any interim time between the end of an event and the start of subsequent event was registered as “waste” time. Events unrelated to blood culture processing were not included in the direct observations.

### Retrospective data retrieval

Data from laboratory information management systems (LIMS) and blood culture incubators were retrospectively extracted to provide a complementary data source from which probability distributions associated with timed events could be inferred. Data were manually extracted for a random subset of positive specimens.

### Quantitative statistical analysis

Fitter (15, 16) was used to fit probability distributions to event-time data, using maximum likelihood estimation. We limited our search to the exponential, gamma, lognormal, Weibull, and uniform distributions due to their applicability in the modeling and simulation of service times (17). The sum of square errors and Kolmogorov–Smirnov test were used to compare distribution fits. The best fitting distribution was selected after further analysis of plausible candidate distributions using Cullen and Frey graphs. Where there were insufficient data points to estimate the statistical moments of the probability distributions, the uniform distribution, which corresponds to the maximum entropy for this scenario, was used (18).

To assess specimen-level correlation in event durations, Pearson’s correlation coefficient (*r*) was calculated for retrospective event times.

### Building a simulator

A discrete event simulation (DES) of the blood culture laboratory processes was constructed in the Python programming language (version 3.11); salabim (version 23.3) was used to control the simulation (19, 20).

The simulator took in a set of input parameters such as blood culture positivity rate and incubation time-to-positivity (see Table S1 for full list). The output was a list of blood culture specimens with associated timestamps for each event (e.g., time received in the laboratory, time entered the incubator, time Gram stain reported, etc.). Simulated specimens were generated with an inter-arrival time drawn from an exponential distribution, such that the rate of specimen generation matched the real rate observed in the study laboratory.

Blood culture specimens were simulated from the time of specimen generation (equivalent to the time of collection) to the reporting of antimicrobial susceptibility testing or completion of 5 days of incubation, for specimens with growth or no growth respectively. Within the simulator, virtual staff members performed work on the specimens, as informed by the process maps.

Since this study did not gather data around staff behavior or actions not related to blood culture processing, a placeholder staff state was required that would encapsulate staff time spent not working on specimens within the simulation. The model was designed to place staff in an “OnHold” wait state when they finished working on a batch of specimens. Staff remained in this dormant “OnHold” wait state for a fixed period of time (provided as an input parameter to the model—“OnHold” wait time in minutes). At the end of the “OnHold” wait time, staff continued working on specimens if there was outstanding work; otherwise, they re-entered the “OnHold” wait state and the loop was repeated.

To identify a plausible value for the “OnHold” wait time, a grid search was used to realize a maximum likelihood estimate of the parameters. First, to compare simulated specimens with those observed in our real data set, we identified four key nodes: (i) receipt to load onto incubator; (ii) receipt to growth detection; (iii) receipt to unload from incubator; (iv) receipt to reporting of Gram stain.

Within the real data set, these four intervals were collected through retrospective data analysis; whereas, the data that informed probability distributions for the simulator were directly observed.

Subsequently, the “OnHold” wait times for all three staff members were varied together at 10 equally spaced intervals from 1 to 100 min. At each step, the simulation was run, and simulated specimens were sampled without replacement to produce the same number of simulated specimens as real specimens. We compared the simulated specimens’ four key time intervals to those of the real specimens by plotting the density of the time intervals. The “OnHold” wait time that produced simulated specimens that most closely matched the real data was chosen as a plausible “OnHold” wait time.

### Sensitivity analysis

To understand the effect of changes in staff availability on output, a single-parameter local sensitivity analysis was performed (21). The “OnHold” wait time for an individual staff member was varied at evenly spaced steps from 1 to 100 min, keeping the other staff members’ “OnHold” fixed at the value identified using the method described above. At each step, the simulation was stochastically repeated 10 times using random number generators. The summary statistic (mean) was also calculated from the stochastic repetitions of the simulation at each step of the sensitivity analysis. This process was repeated for the rest of the staff members.

## RESULTS

### Qualitative data

**Fig 1:**
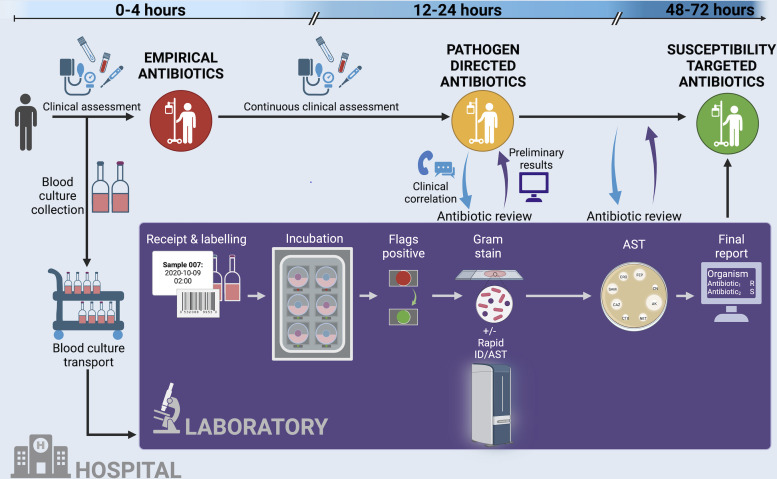
The processing of blood cultures takes place in parallel to the empirical treatment of bloodstream infection and sepsis. At multiple nodes in the clinical pathway, there is an exchange of clinical and laboratory information, leading to the tailoring of antimicrobial therapy to pathogen and susceptibility testing. Our study focused on the events within the inner “Laboratory” (dark purple).

Twelve staff members participated in the observation of blood culture processes, conducted over 19 separate observations. Participants were observed a mean of 2.75 (range 1–5) times. Processes relating to organism identification and antimicrobial susceptibility testing (AST) were all performed in batches. In contrast, none of the processes relating to microscopy and culturing were batched. The complete process map is available in the Fig. S2 to S5. The process map detailed the workings of the laboratory’s role within a wider healthcare environment in which patients are treated for bloodstream infection (Fig. 1).

### Quantitative data

A total of 101 timed observation sessions were performed, covering 13 tasks. Within pre-analytical processes (booking in and loading of samples onto analyzers), 47.8% [range 4.2%–83.4%] of task time was categorized as waste, and in analytical processes (related to positive samples), 31.4% [range 1%–81.3%] was categorized as waste. Table 1 shows the visual fit of the probability distributions against the observational data. The blood culture positivity rate was 11.1%.

**TABLE 1:** Observational data demonstrating statistics obtained for n observations per task, the chosen probability distributions and parameters (data in seconds), and data histogram with probability distribution function overlay (curved line)^*a*^

Task	Obs. (*n*)	Batch size per obs. (mean [range])	Task time min:sec (median [IQR])	Probability distribution	Distribution parameters	Histogram
Label with lab number	17	5.59 [1.00–13.00]	05:14 [2:18–8:36]	Uniform	*a* = 22, *b* = 700	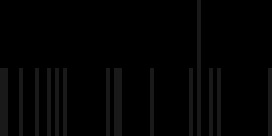
LIMS	17	5.59 [1.00–13.00]	02:56 [1:05–5:42]	Uniform	*a* = 21, *b* = 548	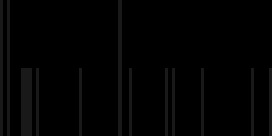
Combine labels	15	3.53 [1.00–7.00]	04:03 [2:35–7:54]	Uniform	*a* = 31, *b* = 838	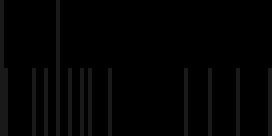
Scan request form	7	4.29 [2.00–7.00]	00:47 [0:40–1:36]	Uniform	*a* = 37, *b* = 109	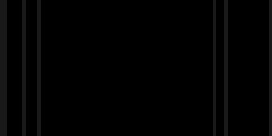
Move location	25	5.24 [1.00–13.00]	00:39 [0:33–0:48]	Lognormal	Mean 3.736, SD 0.385	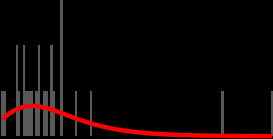
Load onto analyzer	32	4.63 [1.00–13.00]	01:05 [0:34–2:25]	Gamma	Shape 1.493, rate 0.017	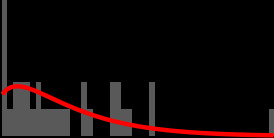
Unload from analyzer	36	1.17 [1.00–3.00]	01:18 [0:47–1:37]	Lognormal	Mean 4.219, SD 0.785	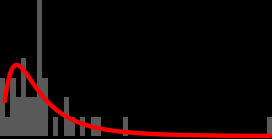
Hazard check	41	1.15 [1.00–3.00]	01:36 [1:00–2:10]	Lognormal	Mean 4.482, SD 0.639	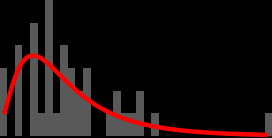
Vent sample	45	1.13 [1.00–3.00]	03:45 [2:47–5:14]	Lognormal	Mean 5.452, SD 0.499	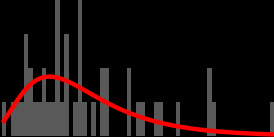
Prepare initial culture/smear	46	1.13 [1.00–3.00]	03:59 [2:23–6:17]	Lognormal	Mean 5.467, SD 0.499	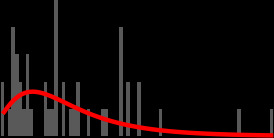
Gram stain smear	46	1.13 [1.00–3.00]	04:41 [3:44–5:23]	Lognormal	Mean 5.595, SD 0.416	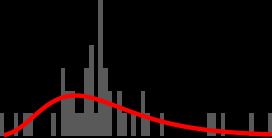
Dry Gram smear	39	1.13 [1.00–3.00]	03:23 [2:09–5:35]	Lognormal	Mean 5.37, SD 0.785	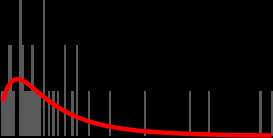
Read & report Gram smear	52	1 [1.00–1.00]	02:46 [1:47–5:31]	Lognormal	Mean 5.185, SD 0.758	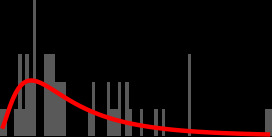

^*a*^ LIMS = loading onto Laboratory Information Management System. Probability distributions are parameterized in minutes.

After excluding specimens with missing data (*n* = 85, 2.8%) from the initial data set of 3,011, retrospective event timing data were available for 2,926 specimens. Of these, 1,523 (52.1%) were excluded because the time of loading onto the analyzer predated receipt in the laboratory. Of the remaining 1,403 samples, 34 had a missing date of collection (2.4%) and were not used for the collection to receipt time analysis. The remaining 1,369 (45.5%) samples had complete data available. Broadly, events showed low levels of specimen-level correlation, supporting the sampling of simulation events from independent probability distributions (see Fig. S6). Table 2 shows the visual fit of the probability distributions against the retrospective data.

**TABLE 2:** Probability distribution fits for retrospectively collected data. Histogram shows observed data with the chosen probability distributions (curved line), data in minutes^*a*^

Process	Collection method	Obs. (*n*)	Median	Probability distribution	Parameters	Histogram
Collection—receipt	Data pull	1,369	2.77 h	Gamma	Shape 0.99, rate 0.003	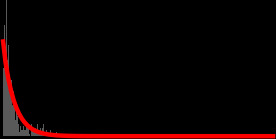
Receipt—load incubator	Data pull	1,403	10 min	Gamma	Shape 0.542, rate 0.015	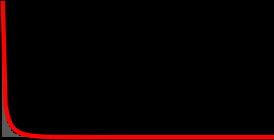
Load incubator—flag positive	Data pull	2,926	16.07 h	Weibull	Shape 1.383, scale 1417	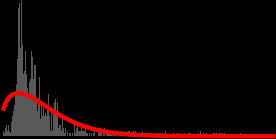
Flag positive—unload	Data pull	2,926	13 min	Weibull	Shape 0.59, scale 23.796	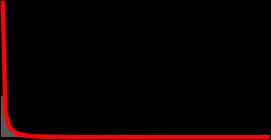
Unload—Gram stain	Manual audit	314	1.18 h	Gamma	Shape 1.665, rate 0.017	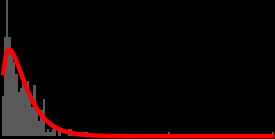
Unload—Gram stain (hub)	Manual audit	205	46 min	Gamma	Shape 1.837, rate 0.028	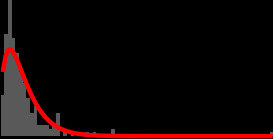
Unload—Gram stain (satellite)	Manual audit	109	2.2 h	Gamma	Shape 4.029, rate 0.026	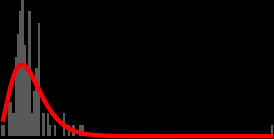
Gram stain—24 h report	Manual audit	297	23.27 h	Lognormal	Mean 7.228, SD 0.221	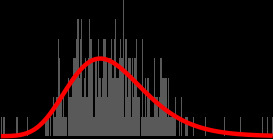
24 h report—48 h report	Manual audit	97	1.03 days	Lognormal	Mean 7.282, SD 0.109	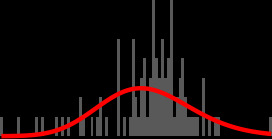
48 h report—final report	Manual audit	60	1.46 days	Lognormal	Mean 7.859, SD 0.772	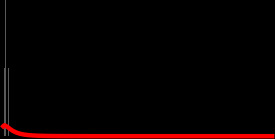

^*a*^ Histogram shows observed data with the chosen probability distributions (curved line), data in minutes. Time period Unload - Gram stain is additionally provided split by site of specimen receipt (hub or satellite). Probability distributions are parameterized in minutes.

### Laboratory simulation

Within the laboratory simulator, blood culture specimens were generated at random intervals and virtually sent to a specimen reception. Three staff agents were simulated: (i) laboratory technicians based in specimen reception; (ii) laboratory technicians working on the blood culture bench; and (iii) laboratory scientists working on the blood culture bench (see Fig. S7). The technicians and scientists working on blood cultures are always available, while the specimen reception technician goes off shift between 20:00 and 08:00. Staff can work on multiple specimens at the same time. After completing jobs within the simulation, staff wait (OnHold) for a period prior to resuming another batch of jobs. This wait represents activities outside the scope of the simulation, such as work on non-blood culture specimens, administrative work, or breaks.

**Fig 2:**
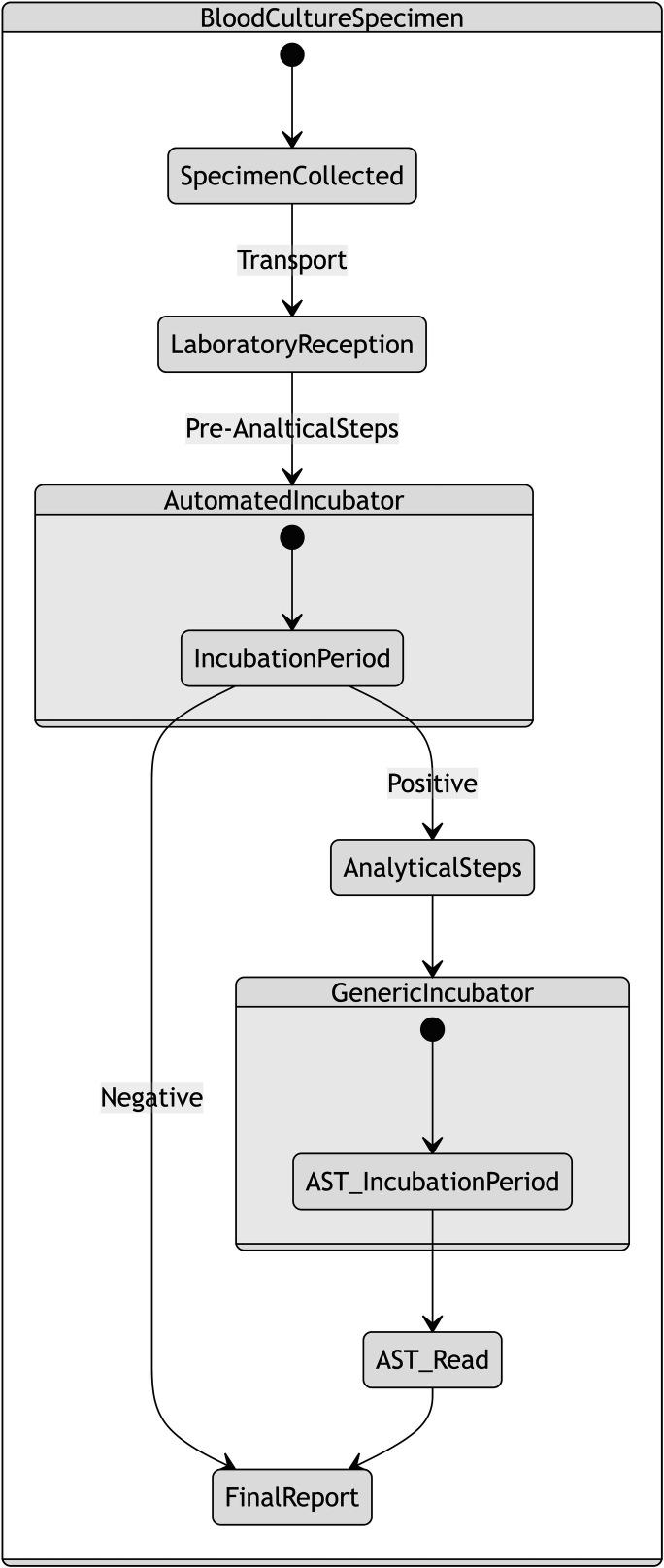
High-level pathway for processing of blood culture specimens within the simulation. AST, antimicrobial susceptibility testing.

Blood culture specimens progressed through the simulation by actions performed on them by staff. The pathway that an individual specimen followed was dependent on whether it had bacterial growth, as shown in Fig. 2. Non-analytical steps were predominantly performed by technicians, whereas analytical steps were predominantly performed by scientists. Specimens were simulated from the time of generation (corresponding to collection from a patient), to the final report (for negative specimens) or antimicrobial susceptibility report (for positive specimens).

We identified 23 min (for all three staff members) as a plausible “OnHold” wait time. When this parameter value was used as an input, the simulation produced simulated specimens that progressed through the four key event nodes at similar rates to the real specimens (see Fig. S8).

**Fig 3:**
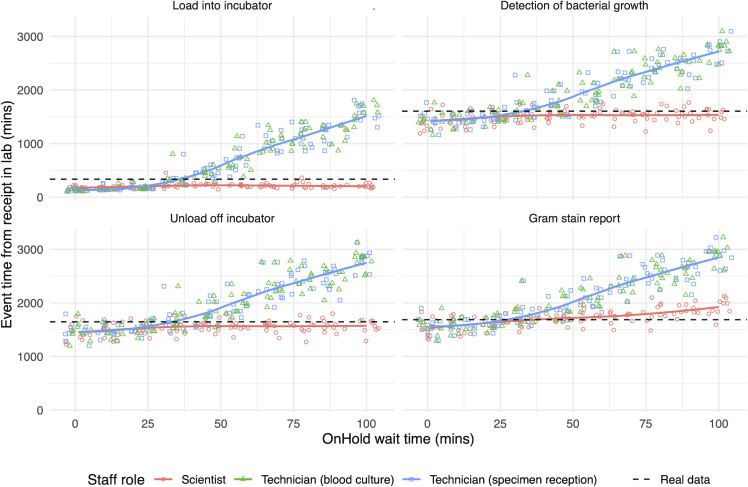
Laboratory simulation sensitivity analysis. Panels show the time from receipt in the laboratory to four key events in the specimen journey. Each dot represents the mean blood culture event time generated from a unique laboratory simulation. All parameters are kept fixed, except for one staff’s “OnHold” wait time (*x*-axis), indicated by the dot color. Colored lines show the aggregate mean of the simulations. The black horizontal line is the mean event times observed in the real data set. Technicians have roles in earlier processing steps; reduced availability cascades delays in all event times. Scientists have roles in later interpretative steps; delays only have an impact on the Gram stain step.

To illustrate potential uses of our model, we considered the sensitivity to changes in staff “OnHold” wait times, and these results are shown in Fig. 3. As “OnHold” wait times increased, a trend toward longer event times (i.e., longer specimen turn-around times) was observed, as seen in Fig. 3. Therefore, the “OnHold” wait time acted as a surrogate for staff availability in our model—higher values constrained staff availability, while lower values freed up staff to work on specimens. This is compatible with model’s design since simulated staff in the “OnHold” state do not perform any work on the simulated specimens (see Fig. S7). Specimen progression through the four key nodes slowed down as the availability of laboratory technicians, who have roles in many steps leading up to Gram stain reporting, decreased. Laboratory scientists’ involvement is in later steps (especially in Gram stain reporting)—correspondingly reducing the scientist availability only had effect on the Gram stain step.

## DISCUSSION

Simulation is extensively used to better understand and optimize manufacturing supply chains, transportation, and military deployment (22–24). Within healthcare, there are multiple examples of queuing, complex supply chains, and multi-step processes that all have a potential impact on clinical outcomes—all of which are ideally suited to be examined through simulation (25). Hence, it is somewhat surprising that simulation techniques are relatively under-utilized in healthcare compared with other industries.

Discrete event simulation (DES) is particularly suited for modeling systems that involve time- and resource-dependent processes and queues (26–28). DES is based on the generation of random events representing time-dependent processes. An advantage of DES is that models are abstractions of real-world processes rather than hypothetical mathematical functions. Therefore, domain experts who are not familiar with the notation of mathematical models may find DES models more intuitively accessible.

In this work, we constructed a DES model of a laboratory system and informed its parameters from a rich data set of retrospective data and directly observed timings. The sensitivity analysis (Fig. 3) revealed three key insights. First, laboratory technicians are key to the timely reporting of blood culture results. Restricting technician availability caused relatively large delays to every key event. Second, these effects were non-linear—the simulated systems coped with reduced technician availability until a progressive backlog of specimens caused the system to decompensate. Finally, delays in early steps within the specimen journey compounded throughout the pathway—although laboratory technicians did not report Gram stains, the event was still sensitive to their availability, because of their roles in preceding steps. Therefore, our results suggest that laboratory technician availability must be preserved, particularly in earlier steps of the pathway, to maintain timely blood culture reports. Laboratory managers can use our model to explore different levels of staff availability and shift patterns and select combinations that avoid the delays seen in the sensitivity analysis.

Our laboratory model can help inform decisions on staffing levels, new testing methodology, and workflow optimization. Furthermore, the model can be integrated within a simulation of the entire patient pathway illustrated in Fig. 1, producing an environment to assess the impact of new interventions in the management of patients with bloodstream infection. “What if?” scenarios can be safely investigated prior to implementation, without any ramifications for patient safety. This approach contrasts with current practices where decisions are made using historical or extraneous data or, worse still, personal beliefs, risking unintended consequences due to the complex and non-linear nature of healthcare systems.

Although the observational component of this study generated detailed process maps and timings for blood culture processing, we did not observe staff’s actions outside of these steps. Therefore, no data were available to inform the simulator’s design with respect to staff non-blood culture actions (i.e., “off time”). To minimize the number of assumptions in this proof-of-concept model, we made a design choice to implement this “off time” as a fixed constant value for all staff members. This approach has important limitations: first, this constant value is an abstraction of what is likely to be a complex collection of sub-processes. Second, the “OnHold” wait time values used in this study cannot be assumed to be appropriate for simulating other laboratories without further calibration. Alternative approaches, such as sampling the “OnHold” wait time from an Exponential distribution, or selecting a different parameter for each staff member, can be explored interactively using our model’s online simulator (https://a-gerada.shinyapps.io/laboratory_simulator/). Furthermore, future work could generate data on staff “off time” (e.g., frequency and duration of breaks or interruptions), which could be incorporated in a future iteration of this model.

Our work provides proof-of-principle that laboratory DES can be constructed and parameterized. While this study is a critical initial step in optimizing a wide range of laboratory and clinical processes, we acknowledge further refinement is required, such as formal parameter optimization using Approximate Bayesian Calibration (ABC) (29). While we used the mean as a measure of central tendency, much of the data were skewed and alternative summary statistics may be better suited for the process of formal parameter calibration (30). Despite any potential limitations, we expect the applications of our research study to be wide-ranging. By adjusting the model’s parameters to new data, our model can be applied to other laboratory settings. Beyond applications in laboratory science, our approach to simulator design can be used to develop similar simulators to seek solutions for other infectious disease clinical challenges that depend on pathway and resource optimization.

## Data Availability

Raw data are available at https://zenodo.org/doi/10.5281/zenodo.10991329. An online version of the simulator is available at https://a-gerada.shinyapps.io/laboratory_simulator/.

## SUPPLEMENTAL MATERIAL

Supplemental material spectrum.01449-24-s0001.docx Fig. S1 to S8; Table S1.

ASM does not own the copyrights to Supplemental Material that may be linked to, or accessed through, an article. The authors have granted ASM a non-exclusive, world-wide license to publish the Supplemental Material files. Please contact the corresponding author directly for reuse.
