# Supplementary material for: Simulation to optimize the laboratory diagnosis of bacteremia
Source: Microbiol Spectr. 2024 Sep 24;12(11):e01449-24. doi: 10.1128/spectrum.01449-24 (PMC11537109; doi:10.1128/spectrum.01449-24)
Supplement: Supplemental material — Fig. S1 to S8; Table S1. [file spectrum.01449-24-s0001.docx]

Supplementary material: Simulation to optimize the laboratory diagnosis of bacteremia

Table of Contents

[Observation form example (Figure S1) 2](#_Toc165470486)

[Simulation input parameters (Table S1) 3](#_Toc165470487)

[Blood culture process maps (Figure S2–Figure S5) 4](#_Toc165470488)

[Supplementary plots (Figure S6–Figure S8) 8](#_Toc165470489)

# Observation form example (Figure S1)


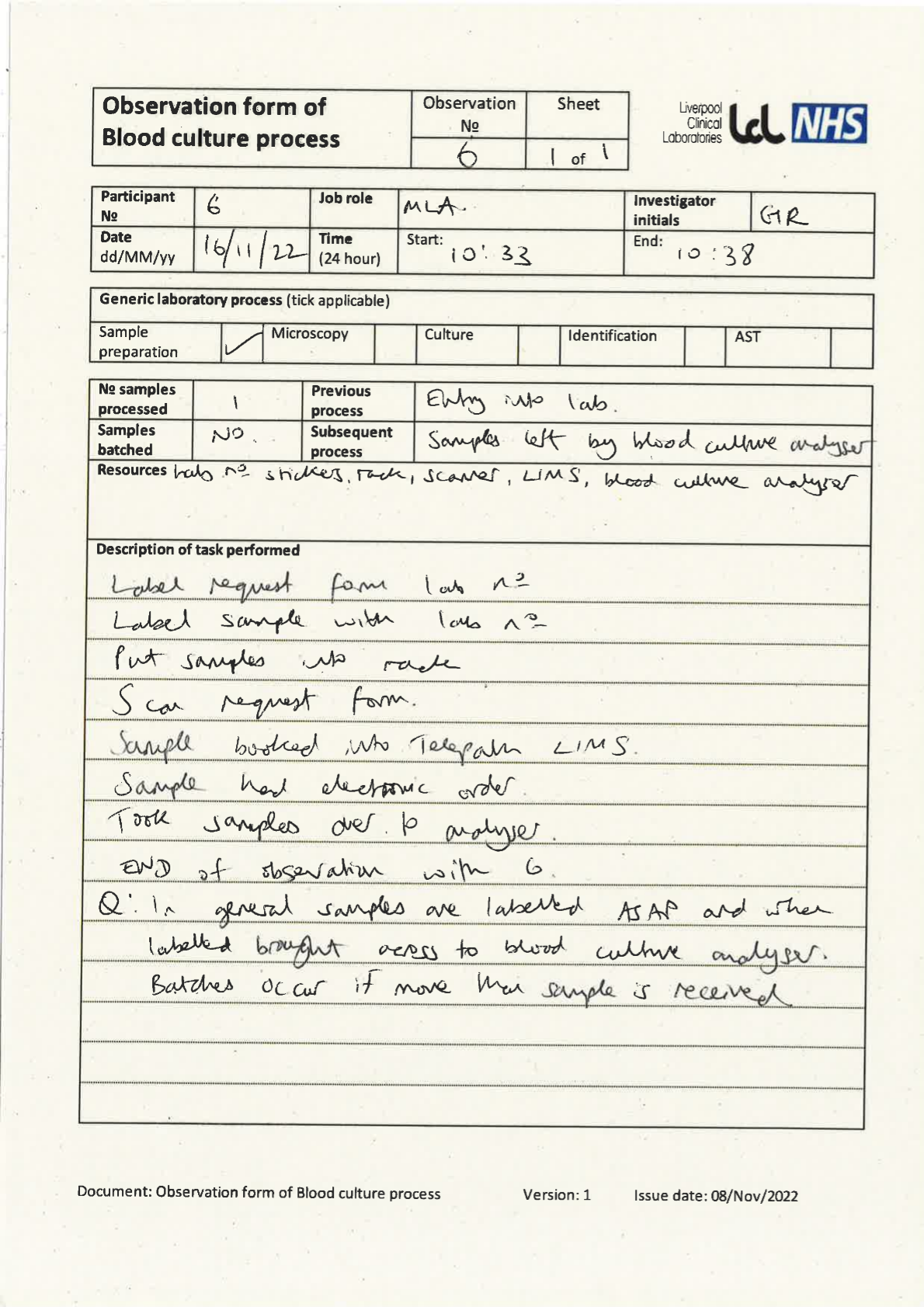


Figure S1 – Example observation form for blood culture processes. LIMS=laboratory information management system, MLA=medical laboratory assistant (technician)

# Simulation input parameters (Table S1)

Table S1: Simulation input parameters not listed in main text.

| **Parameter name** | **Distribution/Value** | **Parameter values** |
| --- | --- | --- |
| Specimen generation rate | Exponential | 0.0638 |
| Shift working on | Boolean | True |
| Shift patterns (reception technician only) |  | 08:00–20:00: On duty  20:00–08:00: Off duty |
| Time to positivity | Weibull | alpha = 1417; beta = 1.383 |
| Maximum incubation time | Constant | 5 days |
| Positivity rate | Proportion | 11.1% |
| Transport time | Gamma | alpha = 0.99; beta = 333.3 |
| AST incubation time* | Constant | 18 hours |
| n specimens per simulation | Constant | 500 |

*AST=antimicrobial susceptibility testing

# Blood culture process maps (Figure S2–Figure S5)

Note: Associate practitioners (AP) were classed as technicians within the simulator.

Figure S2 – Process model of blood cultures from laboratory receipt to processing positive bottle.

Figure S3 – Process model of blood cultures for direct identification from positive bottle using Sepsityper.

Figure S4– Process model of blood cultures for 24-hour culture read.

Figure S5 – Process model of blood cultures for 48 hour and post 48-hour culture reads.

# Supplementary plots (Figure S6–Figure S8)


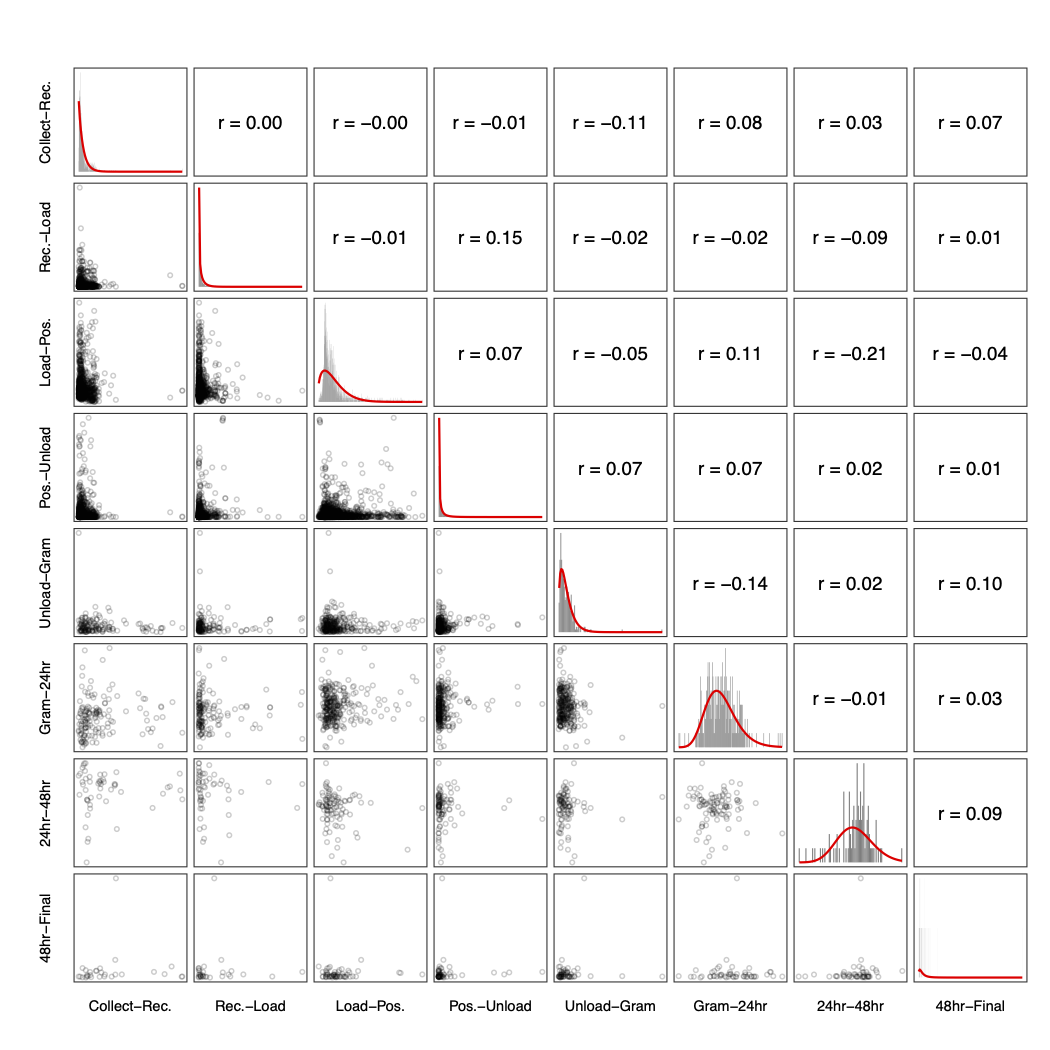


Figure S6 – Correlation matrix for retrospectively collected data. Diagonal blocks show data histograms and the chosen probability distributions. Lower blocks show data points. Upper blocks show Pearson correlation coefficient (r).


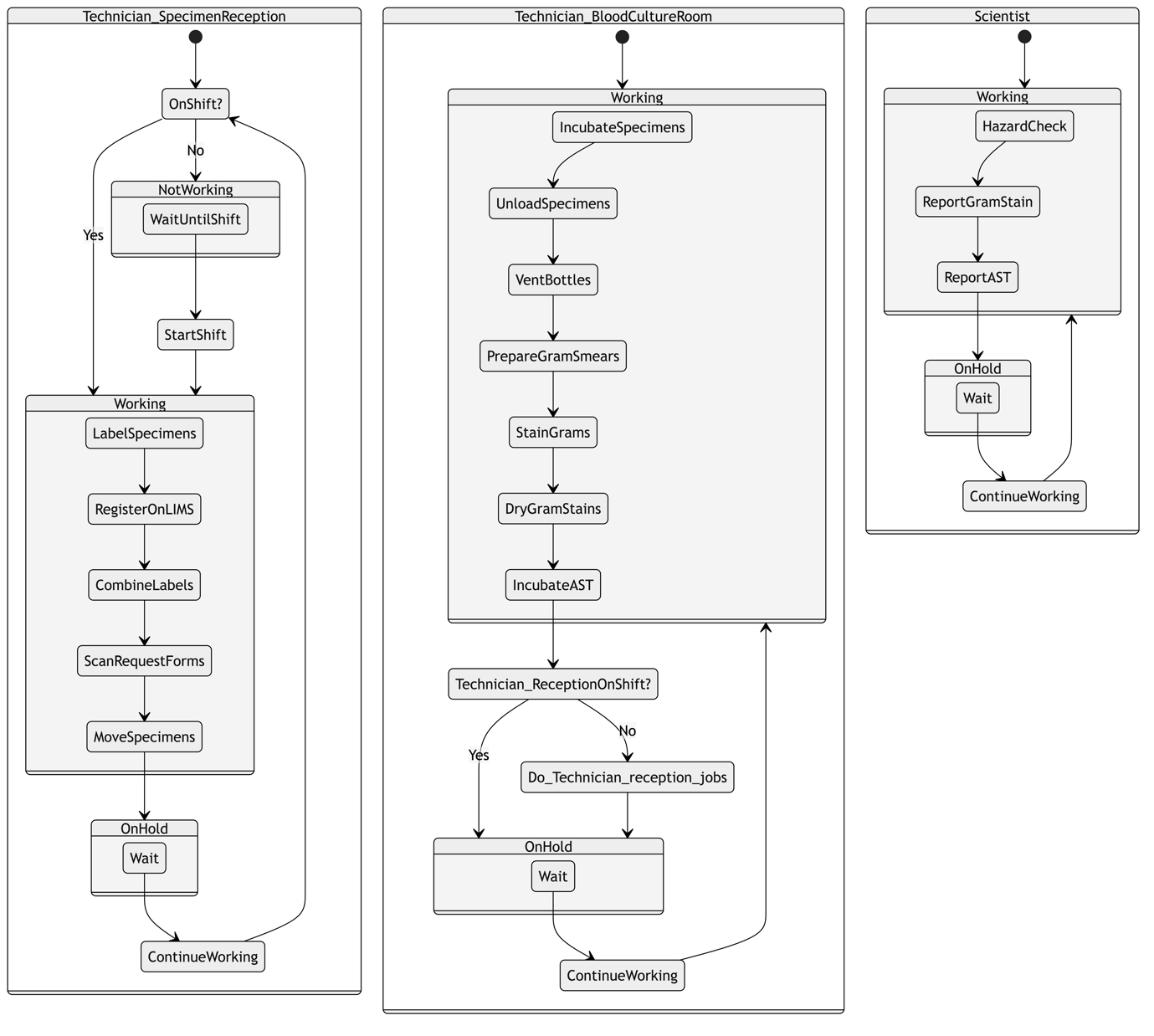


Figure S7 – Behavior of staff within the simulated laboratory. Technicians generally perform processing steps, while scientists generally perform interpretative steps. LIMS = laboratory information management system, AST = antimicrobial susceptibility testing.

Figure S8 – Results of specimens generated from a single simulation run, with distribution of key event node times plotted against those observed in real specimens. 'OnHold' wait time was fixed at 23 minutes for all staff members.
